# Microdochectomy for patients with nipple discharge and the risk of associated breast cancer

**DOI:** 10.1186/s12893-026-03781-8

**Published:** 2026-04-25

**Authors:** Niklas Amann, Zeynep Gökce, Carolin C. Hack, Katharina Seitz, Sophie Eckstein, Manuel Hörner, Carol C Geppert, Arndt Hartmann, Mathias Wetzl, Matthias W. Beckmann, Felix Heindl

**Affiliations:** 1 Department of Gynecology and Obstetrics, Universitätsklinikum Erlangen, Comprehensive Cancer Center Erlangen-EMN (CCC ER-EMN), Erlangen, Germany; 2 Friedrich-Alexander-Universität Erlangen-Nürnberg (FAU), Erlangen, Germany; 3 Bavarian Cancer Research Center (BZKF), Erlangen, Germany; 4 Institute of Pathology, Universitätsklinikum Erlangen, Friedrich-Alexander- Universität Erlangen-Nürnberg (FAU), Erlangen, Germany; 5 Institute of Radiology, Universitätsklinikum Erlangen, Friedrich-Alexander-Universität Erlangen-Nürnberg (FAU), Erlangen, Germany

**Keywords:** Nipple discharge, Microdochectomy, Breast cancer, DCIS

## Abstract

**Background:**

Nipple discharge is one of the most common breast complaints, reported by 80% of women outside the breastfeeding period. It may result from physiological or pathological causes, including intraductal papillomas, ductal carcinoma in situ (DCIS), or invasive breast cancer (BC). This study aimed to evaluate the incidence of malignant upgrades following microdochectomy for nipple discharge and investigate the predictive value of nipple discharge characteristics, in a German population.

**Methods:**

A total of 115 patients with unilateral nipple discharge that underwent microdochectomy from 2019 to 2023 were followed up retrospectively. A standardized diagnostic algorithm was applied, including imaging (mammography, ultrasound), cytological examination of nipple discharge, and histopathological assessment of surgical specimens. We investigated the malignant upgrade after microdochectomy, and the association of different colors of nipple discharge with DCIS and invasive BC.

**Results:**

Histopathology revealed DCIS in 7 cases (6.1%), with concomitant invasive BC in 3 cases (2.6%). Bloody nipple discharge was the most frequent symptom (85.7%) of malignant upgrade cases. However, the rate of malignancy did not significantly differ across discharge types (*p* = 0.442, Fisher’s exact test). All detected invasive BCs were hormone receptor positive, Human epidermal growth factor receptor 2 (HER2/neu) negative, with early-stage tumors (UICC stage IA). Preoperative cytology demonstrated limited sensitivity, with only one of four analyzed malignant cases showing suspicious findings.

**Conclusions:**

Microdochectomy remains a valuable diagnostic and therapeutic tool for patients with unilateral nipple discharge with unsuspicious preoperative breast imaging. The rate of newly detected BC in our study population was low. However, the preoperative identification of patients with increased risk of malignancy stays challenging. Emphasis for future research should be placed on identifying patients with the highest risk for BC e.g. bloody and clear pathologic nipple discharge.

## Introduction

Nipple discharge is one of the most common breast complaints. It is reported by 80% of women at least once in their lifetime outside the breastfeeding period [1]. Nipple discharge can occur physiologically during lactation, as galactorrhea (e.g., in the context of hyperprolactinemia), or pathologically due to benign or malignant breast lesions.

One type of breast lesions that can potentially cause nipple discharge are papillary lesions. Papillary lesions encompass a broad spectrum, ranging from classic intraductal papillomas, with and without atypia, to malignant precursors such as encapsulated papillary ductal carcinoma in situ (DCIS), or finally to the invasive form, known as solid papillary carcinoma [2–4]. Papillomas arise in the milk ducts and present histologically as clearly defined papillary proliferates. They form tree-like branched structures and often present with clinical symptoms such as nipple discharge, which can also be bloody [2]. Papillomas without atypia, diagnosed through interventional breast biopsy, have a low malignant potential of less than 10% [2, 5, 6]. In contrast, the malignancy rate for papillomas with atypia is significantly higher, ranging from 30 to 40% after open surgical removal [5, 6].

DCIS and invasive breast cancer (BC) with no special type (NST) also originate in the milk ducts. In DCIS, carcinoma cells proliferate within the milk ducts without invasion beyond the basal lamina, while invasion into surrounding tissue on the other hand characterizes invasive BC. Invasive BC and especially DCIS can also present with nipple discharge [7].

In patients with nipple discharge, a clinical examination, mammography, and breast ultrasound are performed in daily clinical practice. Additionally, a cytological smear of the nipple discharge is taken to rule out malignant cells in the discharge. It is also recommended to rule out endocrinological causes for nipple discharge (e.g., prolactinoma or thyroid changes). Additional imaging techniques, such as galactography or magnetic resonance imaging (MRI) of the breast, are also used, especially in unclear cases [8].

However, in a portion of patients with nipple discharge, no lesion can be found that is accessible for interventional breast biopsy to rule out malignancy. For these cases international guidelines recommend the surgical removal of the secreting duct – microdochectomy - [9–11]. Previous works reported upgrade rates to DCIS or invasive BC after microdochectomy between 2,3 and 14% [12, 13].

The aims of this study are to evaluate the incidence of invasive BC and DCIS following microdochectomy and to investigate whether presenting symptoms (especially color of nipple discharge) are predictors of malignant upgrade for patients with nipple discharge in a German population.

## Methods

### Study population

**Fig. 1:**
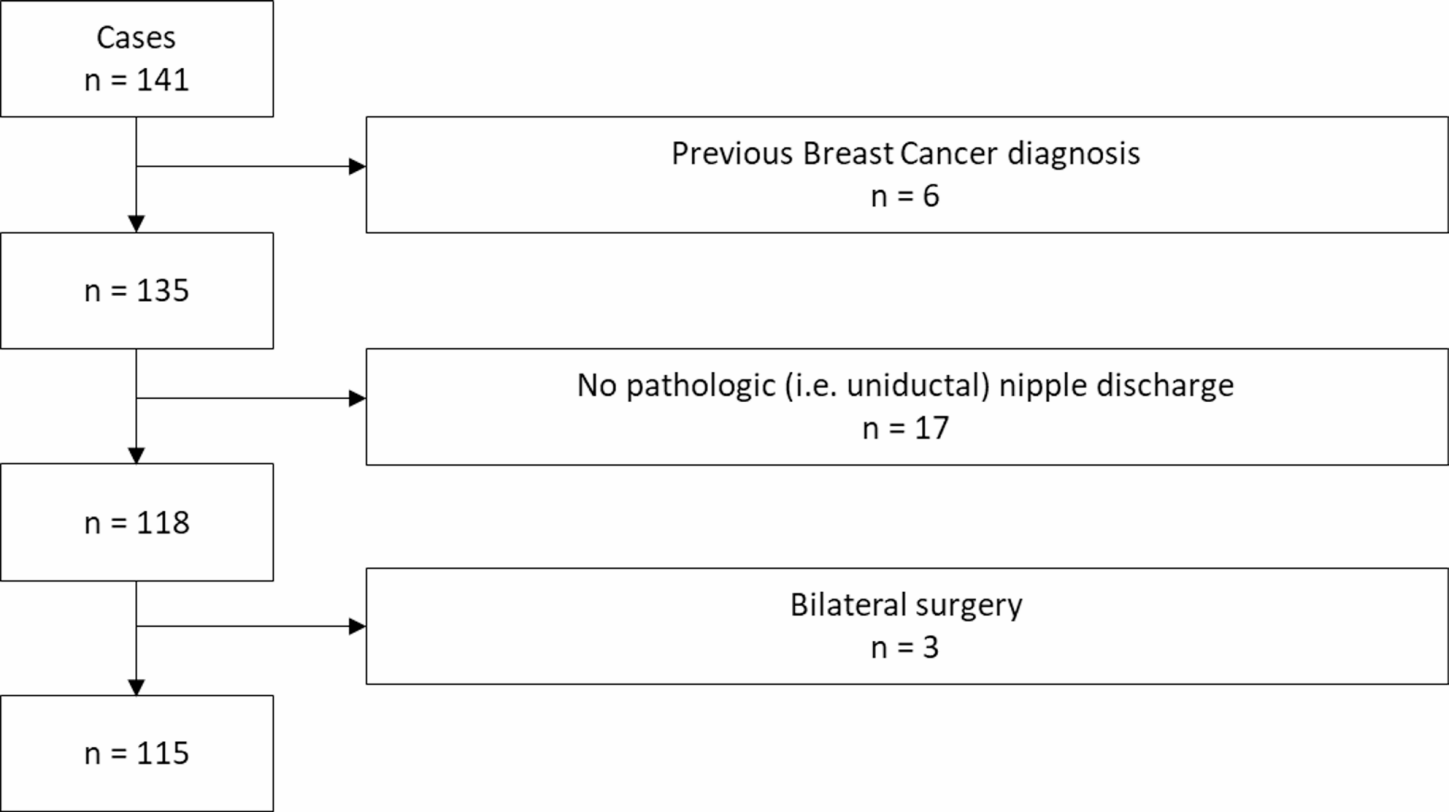
Cases selection

The cases included in this study were retrospectively selected from the database of the Department of Gynecology and Obstetrics, University Hospital Erlangen, Germany. For the period January 2019 until December 2023, a total of 141 consecutive cases were identified that underwent surgical microdochectomy of the breast in the Department of Gynecology and Obstetrics. Six cases were excluded from this analysis because of previous BC diagnosis. Additionally, cases without pathologic nipple discharge (i.e. cases without uniductal nipple discharge, *n* = 17) were removed. Furthermore we excluded cases with bilateral surgery (*n* = 3) from the analysis. The final dataset for this study consisted of 115 patients – see also Fig. 1 for cases selection.

### Diagnostic algorithm for patients with nipple discharge

All patients presenting with nipple discharge in our outpatient-clinic are treated according to a standardized diagnostic algorithm. Following a thorough anamnesis, including a detailed medical history with a focus on medications that could potentially induce nipple discharge (e.g., dopamine agonists), blood samples were collected to rule out endocrinological causes of nipple discharge, such as hyperprolactinemia or hypothyroidism. Using a nipple smear, the discharge was further analyzed by a pathologist at the Institute of Pathology, University Hospital Erlangen, Germany. Subsequently, inspection, palpation, mammography, and breast ultrasound of each breast was performed. In cases with intramammary masses, the correlating lesions underwent interventional breast biopsy. Only cases without lesions in conventional radiological workup and pathologic nipple discharge – i.e. unilateral and uniductal - underwent galactography and subsequently microdochectomy. Galactographies were performed to find underlying intraductal pathologies. In clinical routine it was used to guide the precise direction of surgical resection.

### Microdochectomy

All the microdochectomies were performed at the Department of Gynecology and Obstetrics, University Hospital Erlangen, Germany. The operations were performed by board-certified gynecologists with special training in breast surgery. After standardized preparation including perioperative antibiotic prophylaxis, disinfection, and draping of the surgical site, nipple secretion was digitally provoked. The secreting duct was probed with a blunt microcannula to inject methylene blue. After skin incision of one third of the areola, the blue-marked glandular duct was visually identified and excised into the periphery. After hemostasis and insertion of a drain, the reconstruction of the mammary gland was carried out using local mammary gland rotation flaps. The skin was then closed and a postoperative pressure bandage applied.

### Histopathological workup of the surgical specimens

After fixation of the specimens in formalin for at least 16–24 h, pathological processing and reporting was performed according to current guidelines and the accredited in-house protocol, by board-certified pathologists of the Institute of Pathology, University Hospital Erlangen, Germany. In brief, surgical margins were inked, and the entire FFPE specimen was investigated in step sectioning and if needed further staining, e.g. immunohistochemistry like CK5, ER and Ki-67.

### Histopathological data

All histopathological data were obtained from original reports, which were reviewed by two pathologists. In cases with invasive BC or DCIS during microdochectomy, additional data, including tumor stage, grading, and for invasive BC hormone receptor (HR) status, and HER2 and Ki-67 were investigated.

### Clinical data

Individual histological results from the microdochectomies were obtained for every case from the database of the Institute of Pathology, University Hospital Erlangen, Erlangen, Germany. Basic epidemiological data were extracted from the patients’ records at the Department of Gynecology Erlangen. In cases with invasive BC or DCIS, prognostic and predictive data, including tumor stage, grading, HR, and HER2 status, were extracted from the patients’ records. For all patients, the family history of breast and ovarian cancer according to the German Consortium for Hereditary Breast and Ovarian Cancer was reviewed. Testing criteria are consistent with international guidelines (NCCN, ASCO). The German criteria have a probability of more than 10% for a pathogenic high-risk germline mutation.

### Statistical analysis

Patient and lesion characteristics are described using appropriate summary statistics. Mean and standard deviation are used calculated for continuous characteristics, frequency and percentage for categorical characteristics.

Primary study aim was to investigate the rate of breast malignancy (i.e., resected tissue containing invasive BC and/or DCIS) in patients that had microdochectomy. Furthermore, we investigated whether the preoperative assessment of color of nipple discharge was associated with actual invasive BC or DCIS. For this purpose, the study population was divided into three groups according to the assessment of the color of nipple discharge (i.e., “bloody”, “clear”, and “other”), and for each group the rate of patients with resected tissue containing invasive BC or DCIS was calculated. These rates were compared using the Fisher’s exact test. All analyses were performed using IBM SPSS statistics (Version 29, IBM Corporation, Armonk, New York, USA).

## Results

### Patient characteristics

Between January 2019 and December 2023, a total of 115 female patients underwent microdochectomy for nipple discharge. The patients were referred to the Department of Gynecology and Obstetrics, Erlangen University Hospital, Germany in the case of an abnormal clinical presentation (i.e., nipple discharge). The patients were not recruited due to an abnormal finding in the German National Mammography Screening Program. Only cases without previous BC diagnosis, and unilateral nipple discharge were included in this analysis. Patient characteristics for the overall population are shown in Table 1.

**Table 1:** Patient characteristics

Characteristic		
Age (years)		49.0 (13.7)
BMI (kg/m^2^)		26.4 (6.0)
Inclusion criteria for genetic testing according to the German Consortium for Hereditary Breast and Ovarian Cancer	Negative	110 (95.7)
	Positive	5 (4.3)
Anticoagulation	No	110 (95.7)
	Yes – Acetylsalicylic acid	2 (1.7)
	Yes – Phenprocoumon	3 (2.6)
Color of nipple discharge	Bloody	80 (69.6)
	Amber-colored	2 (1.7)
	Brownish	6 (5.2)
	Yellowish	8 (7.0)
	Greenish	4 (3.5)
	Clear	12 (10.4)
	Milky	3 (2.6)
Preoperative cytology from nipple discharge suspicious for malignancy	No	86 (74.8)
	Yes	4 (3.5)
	Not done	25 (21.7)
Side of microdochectomy and nipple discharge	Left	66 (57.4)
	Right	49 (42.6)
Duration of microdochectomy (minutes)		23.0 (8.7)
Papilloma in histology of surgical specimen	No	48 (41.7)
	Yes	67 (58.3)
DCIS and/or invasive breast cancer in surgical specimen	No	108 (93.9)
	Yes - DCIS	4 (3.5)
	Yes - DCIS and Breast cancer	3 (2.6)

Patient characteristics, showing mean and standard deviation or frequency and percentage

*BMI* Body mass index, *DCIS* Ductal carcinoma in situ

Mean patient age was 49.0 years (standard deviation (SD) 13.7), with very few patients taking anticoagulation therapy (*n* = 5 [4.3%]). Mean duration of surgery was 23 min (SD 8.7). 4.3% of the 115 patients met the inclusion criteria for genetic testing according to the German Consortium for Hereditary Breast and Ovarian Cancer [14–16].

### Histological results from microdochectomy

**Fig. 2:**
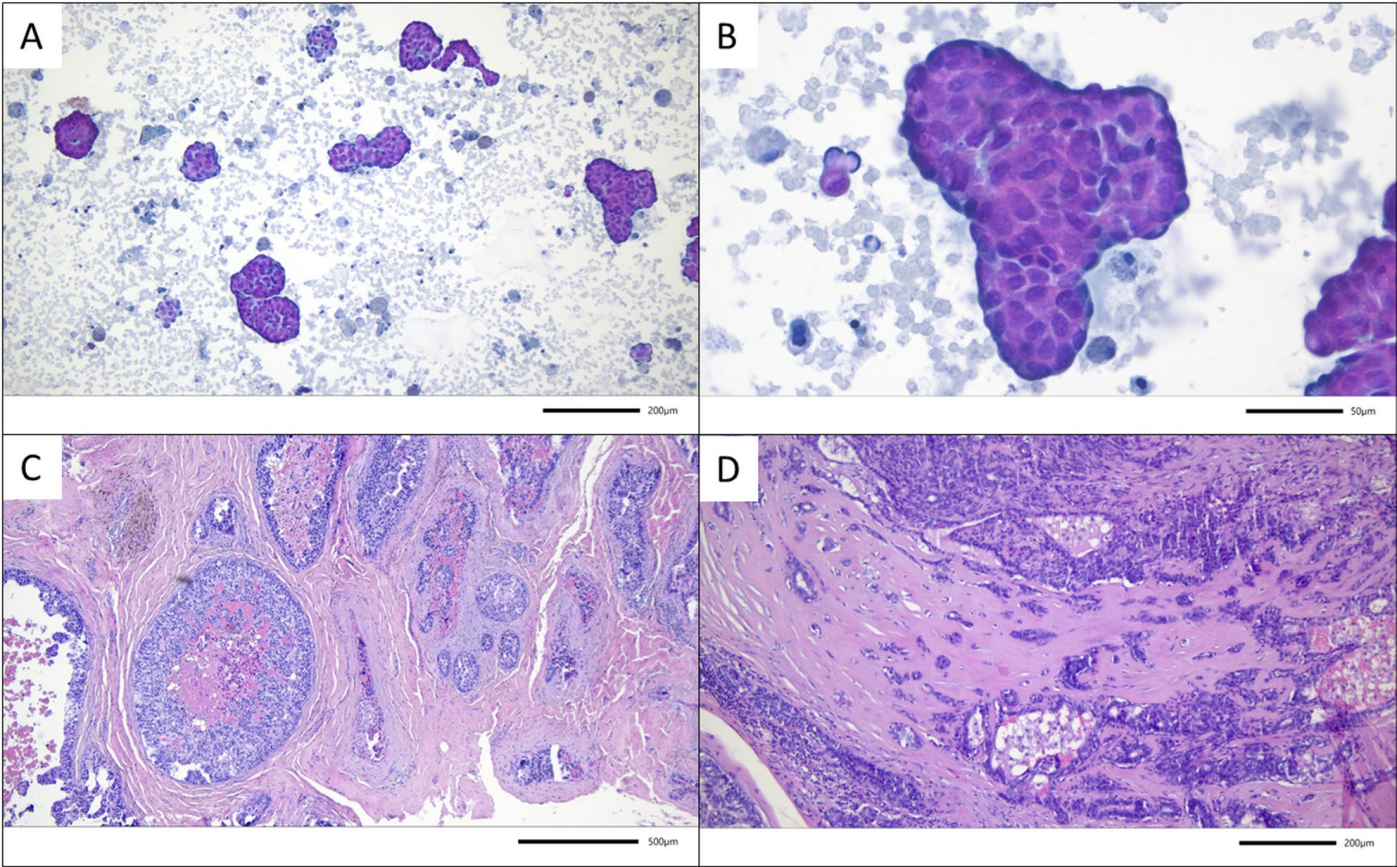
Example for malignant upgrade to DCIS and invasive breast cancer after microdochectomy in case 3. Preoperative cytologic examination of nipple discharge with suspicious cell formations in (**A**) 100x, and (**B**) 400x magnification (Papanicolaou staining). In the surgical specimen DCIS and concomitant invasive breast cancer were found (Hematoxylin and eosin staining, **C**) 25x, and **D**) 100x magnification)

**Fig. 3:**
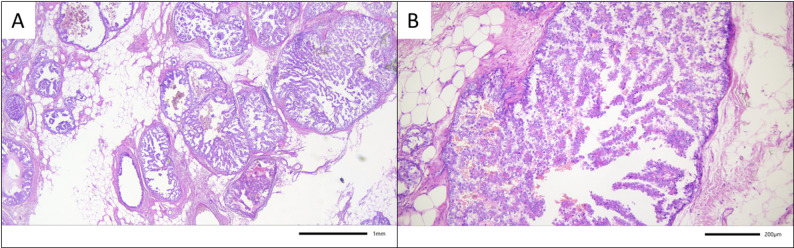
Example for malignant upgrade to DCIS and invasive breast cancer after microdochectomy in case 1. Surgical specimen with a widespread papillary DCIS, extending across the entire specimen and reconstructed to measure up to 6.9 cm, with transition into a 0.6 cm well-differentiated (2 + 2+1 = 5 according to Elston and Ellis) invasive breast cancer of no special type (Hematoxylin and eosin staining, **A**) 25x, and **B**) 100x magnification)

**Fig. 4:**
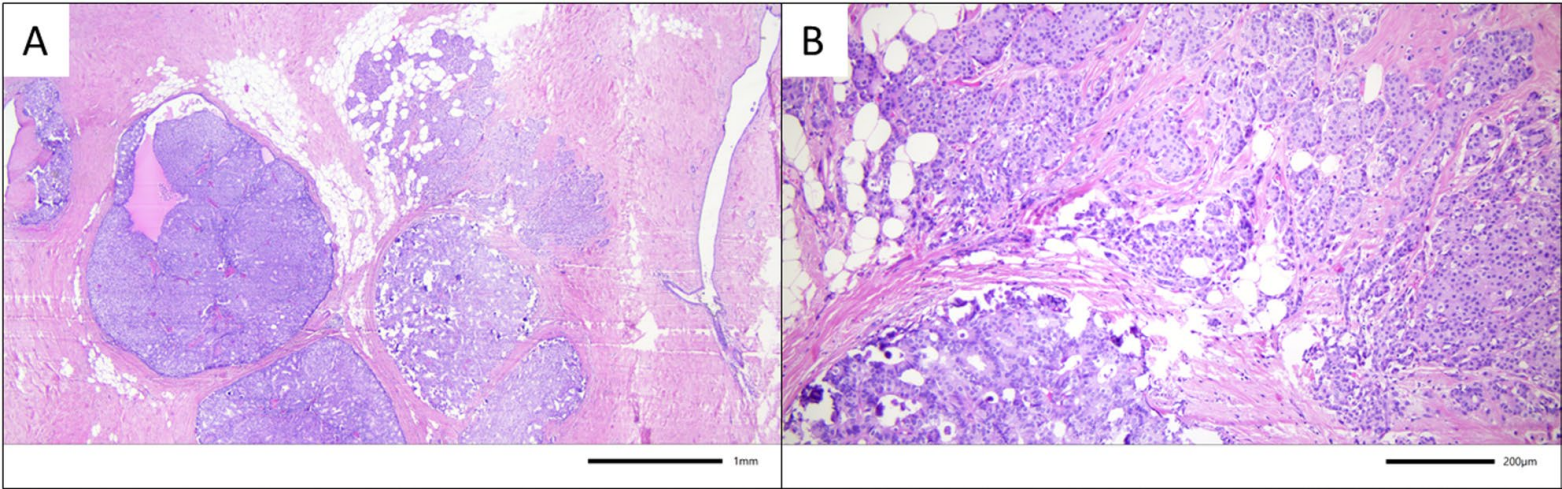
Example for malignant upgrade to DCIS and invasive breast cancer after microdochectomy in case 5. Microdochectomy with focal detection of a 0.4 cm solid papillary DCIS (intermediate nuclear grade) with adjacent 0.3 cm infiltrates of a well-differentiated (3 + 1+1 = 5 according to Elston and Ellis) invasive breast cancer of no special type (Hematoxylin and eosin staining, A) 25x, and B) 100x magnification)

Regarding the histology of the surgical specimens, papillomas were identified after histopathologic workup in 58.3% of all cases. In 7 cases (6.1%) Ductal carcinoma in situ (DCIS) was found in the surgical specimens of microdochectomies. In 3 (2.6%) of those cases, concomitant invasive BC was detected – see Figs. 2, 3, and 4.

For the 7 patients with malignancy in the specimen of microdochectomy, mean age at the time of surgery was 62.4 years (SD 7.7). 85.7% of those patients did not fulfill the criteria for genetic testing according to the German Consortium for Hereditary Breast and Ovarian Cancer. The majority had bloody nipple discharge (*n* = 6, 85.7%). Preoperative cytologic workup of the nipple discharge was done in 4 of the 7 cases. In one case suspicion for malignancy was stated in the preoperative cytology. Table 2 shows the patient characteristics for the cases with and without malignant upgrade.

**Table 2:** Patient characteristics for cases with and without malignant upgrade

Characteristic		No malignant upgrade (*n* = 108)	Malignant upgrade (*n* = 7)
Age (years)		48.1 (13.7)	62.4 (7.7)
BMI (kg/m^2^)		26.3 (6.0)	26.8 (6.4)
Inclusion criteria for genetic testing according to the German Consortium for Hereditary Breast and Ovarian Cancer	Negative	104 (96.3)	6 (85.7)
	Positive	4 (3.7)	1 (14.3)
Anticoagulation	No	105 (97.2)	5 (71.4)
	Yes – Acetylsalicylic acid	1 (0.9)	1 (14.3)
	Yes – Phenprocoumon	2 (1.9)	1 (14.3)
Color of nipple discharge	Bloody	74 (68.5)	6 (85.7)
	Amber-colored	2 (1.9)	0
	Brownish	6 (5.6)	0
	Yellowish	8 (7.4)	0
	Greenish	4 (3.7)	0
	Clear	11 (10.2)	1 (14.3)
	Milky	3 (2.8)	0
Preoperative cytology from nipple discharge suspicious for malignancy	No	83 (76.9)	3 (42.9)
	Yes	3 (2.8)	1 (14.3)
	Not done	22 (20.4)	3 (42.9)
Side of microdochectomy and nipple discharg	Left	60 (55.6)	6 (85.7)
	Right	48 (44.4)	1 (14.3)
Duration of microdochectomy (minutes)		23.1 (8.7)	22.4 (10.7)
Papilloma in histology of surgical specimen	No	42 (38.9)	6 (85.7)
	Yes	66 (61.1)	1 (14.3)

Patient characteristics, showing mean and standard deviation or frequency and percentage

*BMI* Body mass index, *DCIS* Ductal carcinoma in situ.

Tables 3 and 4 provide detailed information on the histopathological findings, including prognostic as well as predictive parameters, for patients with a malignant upgrade to invasive BC or DCIS. Regarding invasive BCs detected, we only found hormone receptor positive, HER2/neu negative, Union for International Cancer Control (UICC) anatomical cancer stage IA tumors. All other cases with malignant upgrade were UICC anatomical cancer stage 0 (DCIS). In total we observed a malignant upgrade rate of 6.1% (7 out of 115 cases) (Table 1).

**Table 3:** Upgrade cases to invasive Breast Cancer after microdochectomy

Case	Patient age	Papilloma in specimen	Breast Cancer type	Tumor size in mm	TNM Stage	Grading/Ki-67	ER / PR / HER2
1 *	62	No	NST	6	pT1b pN0 cM0	G1/ <25%	> 90% / > 55% / negative
3 *	56	No	NST	8	pT1b pN0 cMx	G2/ 40%	> 80% / < 10% / negative
5 *	69	No	NST	3	pT1a pNx cMx	G1 / 10%	> 90% / > 90% / negative

*NST* No special type, *ER* Estrogen receptor, *PR* Progesterone receptor, *HER2/neu* Human epidermal growth factor receptor 2

* Indicating patients with concomitant invasive breast Cancer and DCIS

**Table 4:** Upgrade cases to DCIS after microdochectomy

Case	Patient age	Papilloma in specimen	DCIS grade	DCIS size in mm
1 *	62	No	High-grade	69
2	59	Yes	Intermediate-grade	6
3 *	56	No	High-grade	41
4	63	No	Low-grade	3
5 *	69	No	Intermediate-grade	4
6	78	No	Low-grade	11
7	65	No	High-grade	29

*DCIS* Ductal carcinoma in situ

* Indicating patients with concomitant invasive breast cancer and DCIS

### Color of nipple discharge and risk of malignancy

The rate of patients with diagnosis of invasive BC or DCIS did not significantly differ between the different groups for color of nipple discharge (*p* = 0.442, Fisher’s exact test). The color of nipple discharge was most frequently bloody in 80 cases (69.6%), while in 12 cases (10.4%) it was characterized as clear. The remaining cases (*n* = 23, 20%) had various other colors (e.g., greenish or milky) – Table 1. In 7.5% (*n* = 6 out of 80) of the cases with bloody, and in 8.3% (*n* = 1 out of 12) with clear nipple discharge invasive BC or DCIS was found in the surgical specimen, respectively. In contrast none of the cases with a discharge other than bloody or clear had a malignant upgrade.

## Discussion

Nipple discharge is one of the most common breast complaints. It can occur physiologically, but also repeatedly in the context of malignant processes. In this study, we examined 115 consecutive women with unilateral nipple secretion that underwent microdochectomy over a period of 5 years. In 7 cases (6.1%), a malignant upgrade was observed via microdochectomy. In all patients with a malignant upgrade, DCIS was detected in histopathology. Additionally, three of these patients had concomitant invasive BC.

Our results are in line with other studies showing comparable malignant upgrade rates. In a retrospective study from South Africa of all patients undergoing microdochectomy for nipple discharge in 7.8% a malignant upgrade was found [17]. Another retrospective study showed a carcinoma in 6.0% after microdochectomy or subareolar exploration for pathological nipple discharge [18]. However, other studies reported lower (2.0% [13]) or higher (13.0% [19]) rates for malignant upgrade after surgical excision in the subareolar region. Since we found a rater high rate of papillomas after microdochectomy (*n* = 67, 58.3%) a comparison with papillomas detected in patients by interventional breast biopsy is quite interesting. In patients with papillomas detected by core needle biopsy of vacuum-assisted biopsy the risk of malignant upgrade is assumed to be just under 13%, although in some cases a risk of up to 50% is described [20–23]. This study showed that despite the lack of histological preoperative clarification, a malignant upgrade is not higher than in the case of preoperatively detected papillomas with atypical lesions. In our population we found papillomas in 1 (14.3%) of the cases with a malignant upgrade.

In our study the immunohistochemical examination showed HR positive, HER2 /neu negative BC in all the 3 cases with invasive BC. We observed no obvious high-grade (grading 3), HER2/neu positive or triple negative BC in our analysis. In addition, all invasive BCs had UICC anatomical cancer stage IA. However, 2 cases showed extensive, concomitant DCIS (in maximum 69 millimeters). The largest DCIS – without concomitant invasive BC cases - was of maximum 29 millimeters extent, respectively. However, none of the other studies reported detailed information regarding specific subtypes and prognostic as well as predictive parameters especially of the invasive BCs detected [13, 17–19, 24–30].

For those patients with a malignant upgrade (*n* = 7), 6 cases (85.7%) presented with bloody nipple discharge, while 1 case (14.3%) showed clear discharge. Preoperative cytologic examination was performed in 4 of those cases, and in only 1 patient (14.3%) the nipple discharge was evaluated as suspicious for underlying malignancy. In contrast to our findings a meta-analysis from 2022, including 45 studies, showed a sensitivity of 62% (95% Confidence interval (CI), 0.53–0.71) and a specificity of 71% (95% CI, 0.57–0.81). The meta-analysis reported an overall malignancy rate for patients with bloody nipple discharge of 58% (95% CI, 0.54–0.60), and a positive predictive value (PPV) of 27% (95% CI, 0.17–0.36) [31]. However, in our work we report a PPV for bloody nipple discharge and underlying malignancy of merely 7.5%. The authors of the meta-analysis also stated that nipple smear cytology has limited diagnostic accuracy [31]. It also depends on expertise in excavating and smearing of samples as well as in the cytology itself. Positive rates may also differ in centers versus hospitals with few cases. Also excavating each quadrant of the breast is described to upgrade precision of pre-surgical methods like cytology and is mainly adressed to fine needle aspiration, imprint, nipple discharge or ductal lavage with cytology each [32]. Nevertheless, we found malignant upgrades only in patients with bloody or clear nipple discharge. Other studies have reported malignant upgrades exclusively in patients with bloody nipple discharge, while another study observed intraductal lesions only In cases with bloody, serosanguinous or clear discharge [17, 24]. Since we did not observe malignant upgrade in patients with nipple discharge that was not bloody or clear (e.g. milky, greenish) it seems justified to omit microdochectomy in patients with nipple discharge other than bloody or clear. Due to the limited number of malignant cases, the analysis is likely underpowered. While a high proportion of malignancies were associated with bloody nipple discharge, this trend did not reach statistical significance, and therefore should be interpreted with caution.

The mean age for patients with malignant upgrade was 62.4 years in our dataset. Other studies have also shown that malignancy is more frequently detected in older patients [12, 17]. Nevertheless, some studies found no difference regarding age in patients with and without malignant upgrade [18].

Especially for patients with bloody nipple discharge, galactography has the potential to increase the accuracy of localizing intraductal lesions [27]. However, galactography is being used more and more cautiously as part of the examination for nipple discharge. Like cytology, sensitivity and specificity are limited for galactography. Magnetic resonance imaging (MRI) examinations are clearly superior to galactography. In a recent meta-analysis, the pooled sensitivity for MRI regarding any abnormality was 92% (95% CI, 85–96%), while for galactography it was 69% (95% CI, 59–78%) (*p* < 0.001) [33]. However, MRI examinations are currently not part of the standard examinations for the clarification of unclear breast findings. Routine diagnostics are carried out using ultrasound and mammography. An additional MRI examination was not examined in this study; therefore, we cannot report the impact of breast MRI examinations for the evaluation of nipple discharge. However, breast MRI has the potential to clarify underlying causes of pathologic nipple discharge, especially when conventional imaging is negative [34, 35]. Therefore, we potentially performed to many operations that could have been avoided or be better targeted during the additional use of breast MRI.We only included patients with unilateral nipple discharge in the analysis. Bilateral secretion is often associated with extramammary causes. In addition to drug-induced secretion, endocrinological diseases such as hyperprolactinemia or thyroid disease can cause nipple discharge. All these extramammary causes often result in bilateral nipple secretion. Therefore, we cannot comment on the malignant upgrade rate for bilateral nipple discharge without extramammary causes.

The study’s limitations are twofold: firstly, data on preoperative cytology is absent for 25 of the 115 cases (21.7%), and secondly, the duration of symptoms prior to microdochectomy is not recorded in the dataset. Since nipple discharge can be manually provoked, some cases potentially have non-pathogenic nipple discharge and may be underestimated for malignancy if unilateral nipple discharge was present. The presented retrospective data originate from a tertiary referral center in a university hospital maybe influencing patient acquisition and the cohort represented.

In brief, the advantage of this analysis is the case only study design with the absence of specific inclusion criteria, the reporting of detailed prognostic and predictive information on cases with malignant upgrade, and presenting comprehensive and perioperative data. As proposed by others, surgical excision of breast lesions can be avoided for lesions with an upgrade rate of less than 2%. This is extrapolated analogously to the BI-RADS category 3, where a possible diagnostic delay is not associated with a worse prognosis [36]. Therefore, we suggest microdochectomy for patients with unilateral nipple discharge after exclusion of extramammary causes.

Prospective studies are necessary to validate our data and define optimal strategies in patients with pathologic nipple discharge. Future research should especially focus on the integration of highly accurate imaging techniques in the pretherapeutic workup, especially breast MRI. Prospective studies with detailed information regarding colour of nipple discharge, epidemiological data, and precise imaging techniques could help to develop personalized risk prediction models in patients with pathologic nipple discharge.

## Conclusion

Microdochectomy is an effective diagnostic and therapeutic tool in identifying malignancy in patients with unilateral nipple discharge. With an overall malignancy rate of 6.1%, no malignancies were detected in cases with non-bloody, non-clear discharge. Histopathology identified only DCIS, and early-stage invasive BC, hormone receptor-positive, HER2 negative and favorable prognosis. Preoperative diagnostic tools like cytology and imaging showed limitations, underscoring the importance of surgical excision in cases with unclear findings. Based on our findings, we recommend microdochectomy for bloody, and clear nipple discharge, particularly in older patients and limited diagnostic accuracy of preoperative cytology.

## Data Availability

Data that support the findings of this study are available from the corresponding author upon reasonable request.

## Abbreviations

BC: Breast cancer
CI: Confidence interval
CK5: Cytokeratin 5 antibody
DCIS: Ductal carcinoma in situ
ER: Estrogen receptor
FFPE: Formalin fixed, paraffin embedded material/tissue
HR: Hormone receptor
HER2: Human epidermal growth factor receptor 2
Ki-67: Ki-67 antibody
MRI: Magnetic resonance imaging
NST: No special type
PPV: Positive predictive value
PR: Progesterone receptor
SD: Standard deviation
UICC: Union for International Cancer Control
